# Immature ovarian teratoma with unusual gliomatosis

**DOI:** 10.1186/1757-2215-6-28

**Published:** 2013-04-16

**Authors:** Ancuta Gheorghisan-Galateanu, Dana Cristina Terzea, Mara Carsote, Catalina Poiana

**Affiliations:** 1 C.I.Parhon National Institute of Endocrinology, 34 Aviatorilor Blvd., Bucharest 011853, Romania; 2 Carol Davila University of Medicine and Pharmacy, 8 Eroii Sanitari Blvd., Bucharest 050474, Romania; 3 V.Babes National Institute of Development and Research, 99- 101 Splaiul Independenţei, Bucharest 050096, Romania

**Keywords:** Ovarian teratoma, Immature teratoma, Gliomatosis peritonei, Glial fibrillary acidic protein

## Abstract

This study aimed to investigate an unusual case of immature ovarian teratoma with onset of mature glial cells implanted on the contralateral ovary, a challenge in the diagnosis of the second ovarian tumor. We report the case of a 31- yr-old woman, who developed at the age of 16 an immature teratoma in the right ovary that was surgically removed. Six years later mature glial implants were present on the left ovary and six months later at the level of peritoneum that relapsed after other six months. The patient suffered three surgical resections after the initial one. Paraffin sections and immunohistochemical examinations using antibodies against glial and neuronal antigens were performed. In the teratoma, the neuroectodermal tissue expressed Glial fibrillary acidic protein (GFAP), S100 protein, Epithelial membrane antigen (EMA) and Cytokeratin 34 beta E12 (Ck34beta E12), wheares the implants expressed only GFAP and S100 protein. The immature teratoma is the rarest type of ovarian teratomas. *Gliomatosis peritonei* is an exceptional finding, expecially with onset on the contralaterally ovary. The implant of the mature glial cells has a high risk of relapse, as seen in our case, thus close follow-up of the patient is necessary.

## Background

The ovarian teratomas are represented by mature, immature, and monodermal (as *struma ovarii*, carcinoid tumors, neural tumors) types. They are considered the most common germ cells neoplasm. Teratomas comprise a number of histologic types of tumors, all of which contain mature or immature tissues of germ cell (pluripotential) origin. The immature teratoma (IT) is the currently preferred term for the malignant ovarian teratoma composed of a mixture of embryonal and adult tissues derived from all three germ layers. Any type of tissue may be represented. The main component is usually neurogenic, but mesodermal elements are also common. According to WHO, IT is defined as a teratoma containing a variable amount of immature embryonal type (generally) neuroectodermal tissue [1]. Tumor grading is based on the amount of immature neuroepithelium presence. Regarding the aggressive profile of the glial cells from an immature teratoma, another phenomenon might be found: *gliomatosis peritonei* (GP), the implant with glial cells.

## Case presentation

**Figure 1:**
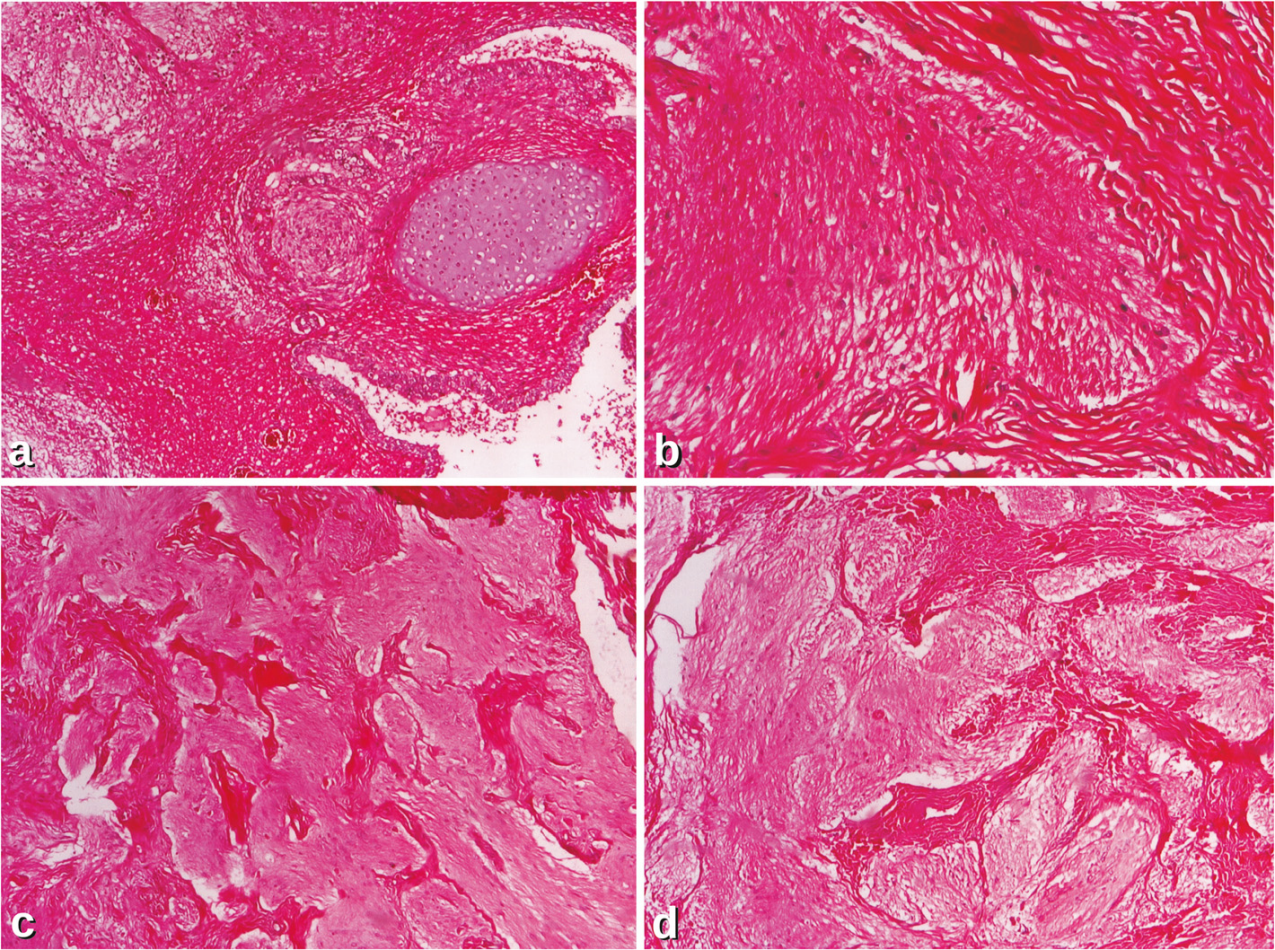
**Microscopic images of resected tumors. a**. Immature teratoma of the right ovary HE stain ×4 **b**. Left ovarian tumor. Ovarian gliomatosis HE stain ×10 **c**. Gliomatosis peritonei (first resection) HE stain ×4 **d**. Gliomatosis peritonei (second resection) HE stain ×4.

**Figure 2:**
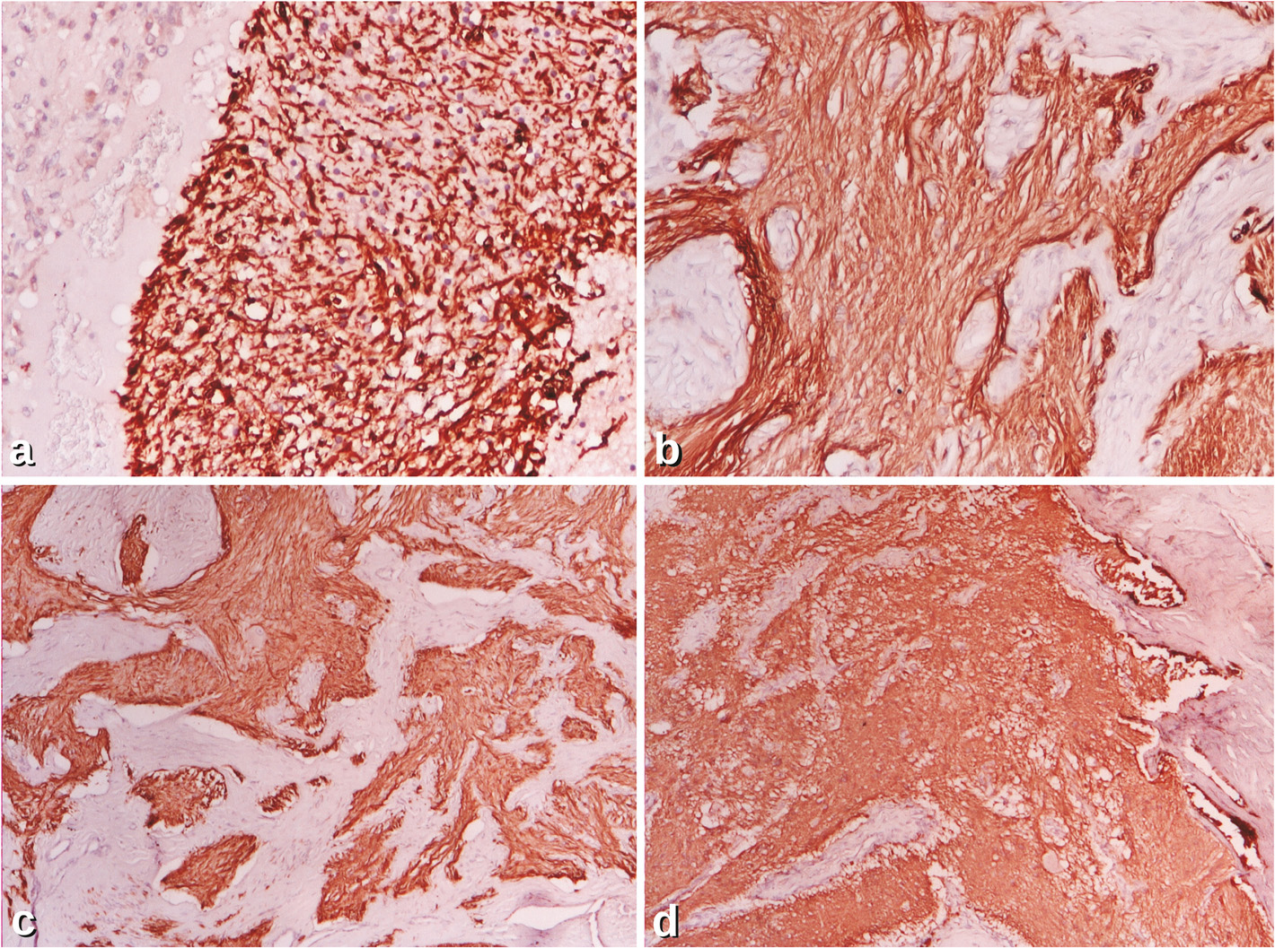
**Glial fibrillary acidic protein (GFAP). a**. Immature right ovarian teratoma ×10 **b**. Mature glial cells in left ovarian gliomatosis ×10 **c**. Mature glial cells in gliomatosis peritonei (first resection) ×4 **d**. Mature glial cells in gliomatosis peritonei (second resection) ×4.

We present a case of a 31 year old female with her medical history. Her family histories were all unremarkable. At the age of 16 she was accidently (at a routine abdominal ultrasound) diagnosed with a right ovarian tumor. The tumor has been removed because of suspected malignant risk. Pathologic examination showed an immature teratoma with glial cells. (Figure 1a) Six years she was asymptomatic then a left ovarian tumor was found because she accused unspecific pelvic pain which was controlled under usual analgesics. The tumor was evidenced by computed tomography and it was completely removed based on intra-operatory decision. The tumor was yellow, large (7 by 6 cm) with solid and multi-cystic aspects associated with another small tumor of 2 cm at the Douglas level. The intra-operatory pathological exam suggested an ovarian possible malignant Sertoli-Leydig tumor that why the ovary was completely removed. Only two years later the pathological exam was re-analyzed and considered a mature glial cells implanted into the ovary. (Figure 1b) Six months later the patient accused persistent abdominal pain and discomfort after meals. The computed tomography showed a tumor of 10 by 6 cm at the Douglas level up to the sigmoid with cystic and solid components, with no evidence of lymph nodes, neither hepatic metastasis. She suffered the tumor resection at the level of peritoneum. At that time the diagnosis of peritoneal carcinomatosis was established and she was treated for six months with chemotherapy (bleomycin, etoposide and cisplatin) when she accused again intense abdominal pain and dysuria, associated with mild elevation of the hepatic enzymes. The forth surgical procedure was performed because it was considered a recurrence: exploratory laparotomy with viscerolysis. This time the pathological exam was re-analyzed for all the surgeries and a mature implant of the teratoma was diagnosed in the second procedure as well as the diagnosis of *gliomatosis peritonei* for the last two procedures was established. (Figure 1c and 1d) The immunohistochemistry profile was also performed at this time and it showed positive staining for glial fibrillary acidic protein (GFAP) in each of the four resections (boths ovaries, as well as peritoneal tumor). (Figure 2) The stains were also positive for S100 protein. The immunohistochemical method was an indirect bistadial technique performed with a polymer based detection system (EnVision^+^ Dual Link System-HRP, Dako, Carpinteria, CA). Only the first tumor, from the right ovary, expressed Epithelial membrane antigen (EMA) and Ck34beta E 12. IT contains different types of cells, including the immature glial cells, while the gliomatosis contains only mature glial cells (Table 1). Since she was ovarectomized, and the malignant diagnosis was finally excluded she came to our attention to evaluate the estro-progestative therapy. No estrogen or progesterone receptors were positive into the tumors so the therapy was started. She was followed-up for the next seven years. We consider that life follow-up is necessary. In addition, at the age of 23 she was diagnosed with primary hypothyroidism and autoimmune thyroiditis, which is currently in substitution treatment with L-thyroxine (75 μg / day). The risk of recurrence in case of gliomatosis persists also the chance of its malignant transformation is low. Based on the medical history, probably an adequate pathology diagnosis at the moment of the second procedure would have saved the second ovary. Yet, the pathological exam is difficult and the pre-operative computed tomography showed a very large tumor. Also, at that moment the immunohistochemistry was not routinely performed. The long medical history of this case highlights that even in rare situation like this the clinical data and its analyses may improve the clinical practice and eventually the patients care.

**Table 1:** The characteristics of the histological and imunohistochemical profile of tumors

	**Immature teratoma**	**Gliomatosis**
Type of glial cells	Immature	Mature
GFAP	+	+
S100 protein	+	+
EMA	+	Negative
Ck 34beta E12	+	Negative

## Discussion and review of the literature

Generally, the teratomas might develop a rather bizarre behavior, such as others ovarian tumors as those with virilizating features due the presence of Sertoli-Leydig cells. The term itself of “teratoma” was derived from the Greek root “*teratos*” which means Monster. The first description of teratoma was made in 1960 by Thürlbeck and Scully [2].

### Teratomas

Teratomas are most frequently found in the gonads (ovary and testes). Extragonadal teratomas are rare and arise from midline structures (thyroid, retroperitoneum, mediastinum, pericardium and brain). Very rarely, teratomas are found in other solid (e.g. breast, parotid gland, liver) and hollow (e.g. oesophagus, stomach, bladder, uterine cervix) organs [3]. Teratomas may be benign, malignant or a component of a mixed germ cell tumor (GCT) [4]. Mature teratomas are benign tumors, which are most often composed of derivatives of two or three germ cell layers. In contrast, immature teratomas are malignant ovarian tumors, as the present case [5]. IT represents 3% of all teratomas, 1% of all ovarian cancers and 20% of malignant ovarian germ cell tumors [6]. It is defined as a tumor containing immature embryo components, usually immature primitive neuroectodermal tissue. Immature elements represent the evolution of a malignant clone, and the prognosis relates to the amount of this component [7]. The lack of 12p amplification in immature ovarian teratomas, in contrast to its presence in other types of malignant ovarian germ cell tumor, demonstrated a different pathogenesis compared to other malignant ovarian germ cell tumors [8]. IT of the ovary is almost always unilateral and is a tumor of children and adolescents, that occurs essentially during the first two decades of life [9]. In our case the incidence was at 16 years of age and also the malignant tumor was unilateral, the contralateral ovary was affected by the process of gliomatosis.

### Gliomatosis peritonei

*Gliomatosis peritonei* (GP) can be defined as the metastatic implantation of glial tissue on surfaces of visceral or parietal peritoneum. GP is a rare situation, characterized by the recurrence of peritoneal implants after the surgical treatment of ovarian teratoma. At histologic analysis, the implants of gliomatosis peritonei resemble benign mature glial tissue with delicate fibrillar processes and scattered supporting cells. Malignant transformation is exceedingly rare [10]. However, some reports have documented the rapid recurrence of immature peritoneal implants, as implantation is associated with teratomas of all grades [11]. GFAP is an intermediate filament protein that expresses with the development of astrocytes in the fetal nerve tissue. GFAP immunostain confirmed the glial nature of the tissue. A strong expression of GFAP often suggests that tumor cells are mature and well differentiated [12]. The association of GP with the immature teratoma is rather classical since the majority of cases with GP are associated with immature ovarian teratoma and extremely rarely found with mature ovarian teratomas [13]*.* In the case we presented, the patient associated ovarian and peritoneal *gliomatosis*, diagnosed six years after the initial removal of the immature teratoma. Paradoxically, patients who have immature ovarian teratomas in association with mature glial implants appear to have a much better prognosis [14]. However, the presence of a secondary tumor, even with benign cells, complicates the evolution of the disease because repeated surgery is necessary as well as ultrasound or computed tomography follow-up over the time. In our case, the 31 years patient suffered four surgical procedures. The origin of glial implants in GP and the factors responsible for the development of GP in association with teratomas has been the subject of many studies.

### Mechanism of implantation

The mechanism of implantation is unknown and two hypotheses to explain the origin of GP have been proposed. The first hypothesis suggests that GP is genetically related to the associated teratoma, and the cells from the primary tumor relocated through a spontaneous or surgical capsular defect or disseminated via angiolymphatic channels [15]. Capsular defects have been described in resected teratomas and in some instances, teratomatous tissue has been observed protruding through these defects [16]. In support of lymphatic dissemination, mature glial tissue has been found in mesenteric, para-aortic, and retroperitoneal lymph nodes in association with immature teratomas, in the presence or the absence of GP [17]. The second hypothesis, who has more supporters, suggested that glial foci are genetically unrelated to the associated teratoma, glial implants developing from normal cells in the peritoneum or subjacent mesenchyme. They presumably originate in pluripotent Müllerian stem cells, which have undergone a metaplastic process in response to an unknown neoplastic stimulus [18]. The implant cell needs a favorable matrix environment in order to survive.

### Associated or independent?

To determine whether glial implants are genetically related to the associated ovarian teratoma or whether they arise independently, it was studied the genetic profile of ovarian teratomas. Approximately 65% of teratomas are derived from a single germ cell after the first meiotic division with subsequent failure of meiosis 2 or endoreduplication of a haploid ovum [19]. Molecular studies suggest that ovarian teratoma and GP are genetically distinct (multiple independent tumors rather than relapse or metastasis). Some studies have demonstrated that all implants and normal tissue showed heterozygosity at each of the three microsatellite loci on different chromosomes, whereas teratoma showed homozygosity at same microsatellite loci, indicating that glial implants in GP often arise from cells the peritoneum, and not associated with ovarian teratoma [20]. Another study concluded that GP is probably derived from metaplasia of submesothelial cells [21]. The remarkable ability of stem cells derived from various organs to differentiate along divergent pathways has been the subject of several articles, including studies demonstrating that bone marrow-derived stem cells can undergo glial differentiation [22]. A stem cell’s microenvironment can induce a specific differentiation pathway, and it is possible that some teratomas with an abundant glial component secrete factors that induce glial differentiation in the peritoneum. Murine astrocyte cells and teratocarcinoma cell lines have been shown to secrete β-nerve growth factor *in vitro*[23]. GP has been described in children without teratomas who have had ventriculo-peritoneal shunts placed early in infancy. In this case, neural growth factors, normally present in cerebrospinal fluid, may enter the peritoneum through the shunt and induce glial differentiation in the same manner [24].

### Treatment and prognosis

The treatment of IT and GP is complete surgical resection, also useful for identifying the presence or absence of malignant lesions and for preventing malignancy transformation of the GP residual fragments. Because the lesions are extensive, complete excision is usually very difficult. Potential for recurrence is high, and therefore requires a careful monitoring of residual lesions using scanning imaging such as computed tomography. In our case, the diagnosis of GP was difficult since it started as a contralateral ovarian tumor diagnosed with MRI. The prognosis of IT heavily depends on the FIGO stage [25]. It is influenced by several factors, such as tumor grade, growth pattern, capsular rupture and vascular invasion. It is important to separate from this group the teratomas that also have a yolk sac tumor pattern, since the prognosis substantially decreases under these circumstances [26].

Recent study indicates that Oct4 might serve as a promising biomarker for the diagnosis of highly malignant cases of immature teratoma because Oct4 expression was exclusively detected in immature neuroepithelium of high-grade immature teratomas [27]. Immature ovarian teratoma usually shows only relatively minor cytogenetic abnormalities, in contrast to the other forms of malignant ovarian germ cell tumors, with increasing abnormalities as the grade of the immaturity becomes higher [28]. Some authors believe that the histological grade could be correlated to the presence of aneuploidy. Indeed, immature teratomas grade 1 and 2 are diploid in 90% of cases, while most teratomas grade 3 are aneuploid (66% of cases). In addition, karyotype abnormalities are more frequent in grade 3 [29].

## Conclusion

Immature teratoma is a malignant tumor of the first decades of life. In some cases, the difficult evolution comes from the association with glial implants, as seen in our case at the contralateral ovary, and the peritoneal level. The glial features are reflected by positive GFAP and S100 protein in gliomatosis, as well as in the primary tumor. In patients with immature teratoma and no evidence of disease recurrence, surgery alone is an effective treatment of the primary tumor. In immature teratoma associated with gliomatosis peritonei, combined chemotherapy is recommended. Surgery and chemotherapy can give longer survival even in recurrent disease. MRI may also have a role in monitoring patients for many years as a safe, reproducible and accurate technique for patients who associated GP.

## Consent

Written informed consent was obtained from the patient for publication of this Case report and any accompanying images. A copy of the written consent is available for review by the Editor-in-Chief of this journal.

## Competing interests

The authors declare that they have no competing interest.

## Authors’ contributions

AGG: have made analysis and interpretation of data, analysis and microscopic image processing in final form for publication; have been involved in drafting the manuscript and revising it critically for important intellectual content; have given final approval of the version to be published. DCT: have made paraffin sections and immunohistochemical examinations, pathological diagnosis and photo mycroscop. MC: have made acquisition and analysis of data; have been involved in drafting the manuscript. CP: have made the design of the study, analysis and interpretation of data and revising the study critically for important intellectual content. All authors read and approved the final manuscript.
